# Patients’ and Health Care Workers’ Perception of Migraine Images on the Internet: Cross-sectional Survey Study

**DOI:** 10.2196/32707

**Published:** 2021-11-12

**Authors:** Bianca Raffaelli, Pia Kull, Jasper Mecklenburg, Lucas Hendrik Overeem, Elisabeth Storch, Maria Terhart, Lars Neeb, Uwe Reuter

**Affiliations:** 1 Department of Neurology Charité - Universitätsmedizin Berlin Berlin Germany; 2 Universitätsmedizin Greifswald Greifswald Germany

**Keywords:** migraine, stigma, mass media, stock photos, advocacy, internet, perception, headache, pain, cross-sectional, survey, stereotype, media, awareness

## Abstract

**Background:**

The representation of migraine in the media is stereotypical. Standard images of migraine attacks display stylish young women holding their head in a pain pose. This representation may contribute to the social stigmatization of patients with migraine.

**Objective:**

We aimed to analyze how patients with migraine and health care workers perceive online images of migraine.

**Methods:**

The study consisted of an anonymous web-based survey of patients with migraine at the Headache Center of Charité – Universitätsmedizin Berlin (migraine group) and employees and students at our university (health care group). A total of 10 frequently used Adobe Stock photos of migraine attacks were presented to the participants. Each photo was rated on a scale of 0% to 100% based on how closely it resembled a realistic migraine attack (realism score). Patients with migraine also indicated how much each photo corresponded to their own experience of migraine as a percentage (representation score). We calculated the mean realism and representation scores for all photos and conducted further analyses using the categories male or female models, younger or older models, and unilateral or bilateral pain pose.

**Results:**

A total of 367 patients with migraine and 331 health care employees and students completed the survey. In both groups, the mean realism score was <50% (migraine group: 47.8%, SD 18.3%; health care group: 46.0%, SD 16.2%). Patients with migraine identified their own migraine experience in these photos to a lesser degree (mean representation score 44.4%, SD 19.8%; *P*<.001 when compared to the realism score). Patients and health care workers considered photos with male models to be more realistic than photos with females (*P*<.001) and photos with older models to be more realistic than those with younger people (*P<*.001). In the health care group only, a bilateral pain posture was deemed more realistic than a unilateral pose (*P*<.001).

**Conclusions:**

Standard images of migraine attacks are considered only slightly or moderately realistic by patients and health care workers. Some characteristics perceived as more realistic such as male sex or older age are in contrast with migraine epidemiology. A more accurate representation of migraine in the media could help to raise awareness for migraine and reduce the associated stigma.

## Introduction

Migraine is one of the most common neurological diseases, with a prevalence of 15% in the general population and rising to over 25% in women of childbearing age [1-3]. Migraine causes significant limitations in quality of life and functioning [4]. It is the second most common cause of health impairment among nonfatal diseases worldwide, as shown by the years lived with disability measure [4]. Among individuals aged 15 and 49 years, migraine ranks first among the most disabling of diseases [5].

Despite the substantial impact on patients’ lives, migraine burden is often underestimated [6]. Many patients feel that their symptoms are dismissed as insignificant [7,8]. Platitudes such as “everybody has headaches” or “it's just stress” are omnipresent in the lives of patients with migraine [7,8]. In a survey by Buse et al [9] , almost half of patients with chronic migraine had the impression that their partner did not believe in their disease. The invisibility of migraine can lead to frustration and stigmatization [7]. Out of fear of being doubted, some patients even hide their symptoms and do not seek treatment, which in turn can have a negative impact on the course of the disease [10,11].

The lack of acceptance of migraine as a real disease has historical roots. Until recently, patients with migraine were portrayed as frail women with weak nerves [12]. Although this representation originates from a cultural background different from the present times, these stereotypes continue to shape the common view of patients with this disease [12].

Currently, digital media, and especially the internet, have become an important source of information on health topics [13]. Portrayals of people with migraine in the media can provide an overview on how society currently sees these patients [14]. Most images resulting from the search term “migraine” show slim and stylish young women holding their temples with an expression of pain on their faces [14]. This trivializing and one-sided portrayal could contribute to the insufficient recognition of migraine-related burden and the growth of social stigma [14].

While this stereotypical representation has already raised concerns among experts [14], no study has assessed how the public perceives such images of migraine. In this study, we aimed to investigate the following questions: (1) do patients with migraine and nonaffected health care workers perceive such photos as realistic? and (2) can patients with migraine relate to these portrayals?

## Methods

### Study Design

This anonymous web-based survey was performed on the REDCap (Research Electronic Data Capture) platform. The link to participate in the survey was distributed among the following two groups:

1. The migraine group: patients at the Headache Center, Charité – Universitätsmedizin Berlin, with a diagnosis of migraine in 2020 per International Classification of Headache Disorders–3 (ICHD-3) criteria [15];
2. The health care group: employees and students at the medical school of Charité – Universitätsmedizin Berlin without migraine.

Patients with migraine received the link to participate via a letter in order to comply with data protection law, while the health care group was invited via email distribution lists and social media groups.

The survey structure is illustrated in Table 1. After the assessment of demographic, occupational, and migraine characteristics, 10 different photos of migraine attacks were presented to the participants on the screen. The participants were instructed to rate on a scale between 0% and 100% how much each picture corresponded to a realistic migraine attack. We defined this percentage value as the realism score. Patients with migraine then indicated how closely each image resembled their own migraine experience on the same 0%-to-100% scale. This score was named the representation score.

**Table 1:** Structure of the survey.

Section	Description
Study information	Written information about the study design and aim, as well as the data protection statement agreement. Subjects could download the study information to keep for their records.
Written consent form	In order to access the other questionnaires, participants must confirm that they are ≥18 years of age, that they are voluntarily participating in the survey, and that they agree to the publication of the study results in an anonymous form. Participants could download a consent form to keep for their records.
Demographic characteristics	Participants are asked about their gender, age, ethnic background, height, weight, and highest level of education.
Migraine information	Participants are asked whether they experienced migraine, if they have close family members or friends with migraine, and how they assess the impairment caused by migraine in the general population and in their own migraine experience (on a numerical analog scale from 0% to 100%).
Occupational characteristics	Participants are asked whether they work or study at the Charité – Universitätsmedizin Berlin, in which field, and if they have regular contact with patients with migraine at work.
Photos 1-10	Participants are asked to look at 10 photos of migraine attacks and rate how realistic each photo is. Patients with migraine are also asked how representative of their own migraine experience these photos are.

The photos with models were obtained from the stock photo website Adobe Stock (Adobe Inc) [16]. We purchased a commercial license for the use of the 10 photos in the survey. Image selection was based on the following criteria:

- Result of the search term “migraine”;
- Sorting by the number of times the photos were downloaded;
- 7 females and 3 males (to match the epidemiological sex distribution of migraine);
- Only 1 person in the photo;
- No black-and-white images;
- No heavy editing or heavy filters and effects;
- Face is visible;
- Only 1 photo of each model;
- Person in the foreground (ie, the background does not take more than half of the image).

### Outcomes and Objective

The primary outcomes of the study were the mean realism score for all photos in both groups and the mean representation score for all photos in patients with migraine.

The secondary outcomes were the mean realism and representation scores for the following categories of photos:

- Photos with female models (n=7);
- Photos with male models (n=3);
- Photos with a unilateral pain posture (ie, models holding one side of the head, n=6);
- Photos with a bilateral pain posture (ie, models holding both sides of the head, n=3);
- Photos with younger models (n=5);
- Photos with older models (n=4).

The allocation of the photos into each category was agreed upon unanimously by all authors of this paper. To differentiate between younger and older models, we focused on physical characteristics such as face wrinkles or hair color (ie, white or gray).

### Statistical Analysis

The analysis comprises all participants who rated all 10 photos. Employees or students with self-reported migraine were excluded from the health care group.

Demographic and occupational characteristics, as well as realism and representation scores, were summarized with descriptive statistics (absolute frequencies and percentages for categorical variables and mean (SD) values for numerical variables).

We compared the primary and secondary outcome measures between the migraine and health care groups using independent *t* tests. The realism and representation scores were compared within the migraine group using paired-sample *t* tests.

We further assessed the correlation of the primary and secondary outcomes with sex, age, ethnicity, highest level of education, and occurrence of migraine among family and friends using Pearson correlation analyses. A 2-tailed *P* value ≤.05 was considered statistically significant. *P* values were corrected for multiple comparisons with the Bonferroni method.

### Ethics Approval and Consent to Participate

The study was approved by the ethics committee of the Charité – Universitätsmedizin Berlin (EA1/213/20). Participants were required to provide electronic consent prior to completing the survey.

## Results

### Population

**Figure 1:**
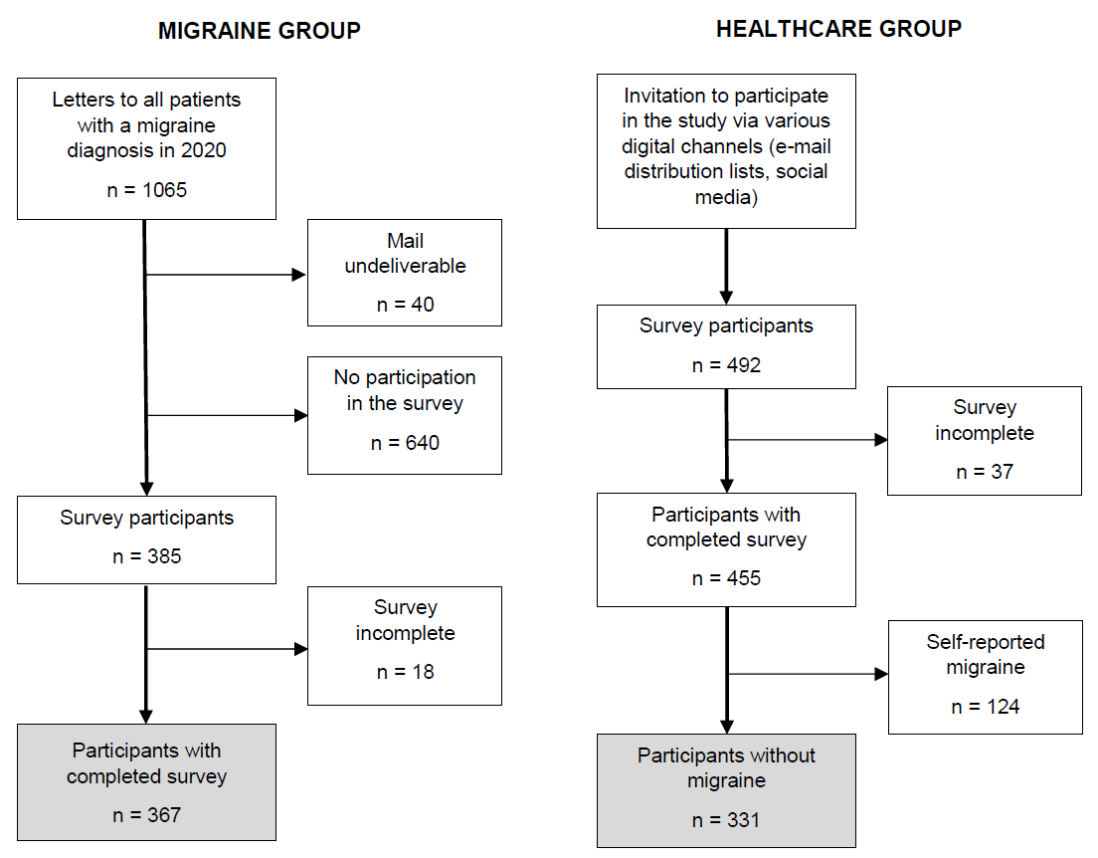
Flowchart of participant selection in both groups.

Between October 27, 2020, and January 15, 2021, 367 patients with migraine and 331 Charité employees and medical students completed the survey. The participant selection process is illustrated in Figure 1.

The majority of patients were female (n=318, 86.6%), with an average age of 45.3 (SD 12.7) years. Participants in the health care group were younger (mean 32.1, SD 11.1 years), but their gender and ethnic distribution was similar to that of the migraine group (Table 2).

**Table 2:** Demographic and occupational characteristics of the survey participants.

Characteristic			Migraine group		Health care group
Age (years), mean (SD)			45.3 (12.7)		32.1 (11.1)
Female sex, n (%)			318 (86.6)		245 (73.9)
Northern or Central European descent, n (%)			314 (85.6)		283 (85.6)
Height (cm), mean (SD)			168.9 (8.1)		171.5 (9.7)
Weight (kg), mean (SD)			69.4 (14.6)		68.3 (13.3)
**Highest level of education, n (%)**					
	University degree	154 (42.1)		118 (35.6)	
	High school diploma	63 (17.2)		155 (46.8)	
	Technical baccalaureate	29 (7.9)		8 (2.4)	
	Apprenticeship	60 (16.4)		20 (6.0)	
	Intermediate secondary school diploma (Realschulabschluss)	38 (10.4)		13 (3.9)	
	General secondary school diploma (Hauptschlussabschluss)	4 (1.1)		2 (0.6)	
	Other	19 (4.9)		15 (4.5)	
Close friends or family members with migraine, n (%)			196 (53.4)		104 (31.4)
**Health care workers’ occupation, n (%)**			—^a^		167 (50.5)
	Physician	—		42 (25.1)	
	Nurse	—		33 (19.8)	
	Other medical professionals	—		27 (16.2)	
	Other nonmedical professionals	—		65 (38.9)	
**Health care students’ study subject, n (%)**			—		164 (49.5)
	Human medicine	—		122 (74.4)	
	Dentistry	—		11 (6.7)	
	Other	—		31 (18.9)	
Regular professional contact with patients with migraine, n (%)			—		68 (20.5)

^a^Not applicable.

### Patients With Migraine

#### Realism Scores

**Figure 2:**
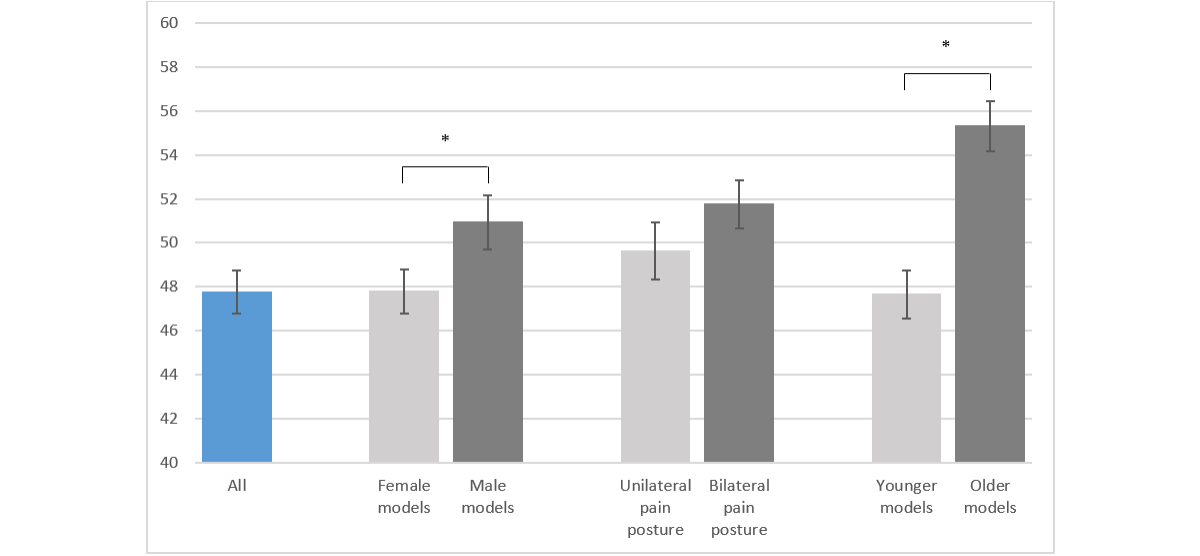
Realism scores of patients with migraine for all photos and different categories. Values are mean (SD), and the asterisk indicates *P*<.001.

Among patients with migraine, the mean realism score for the 10 photos was 47.8% (SD 18.3%). Only 3 out of 10 photos had a mean realism score >50%. Photos with male models were considered more realistic than photos with female models (mean 51.0%, SD 22.4% vs mean 47.8%, SD 18.3%; *P*=.002). Patients rated images with older models as more realistic than those with younger models (mean 55.3%, SD 21.0% vs mean 47.7%, SD 20.1%; *P*<.001). Photos with unilateral and bilateral pain postures had similar realism scores (*P*>.99, Figure 2).

#### Representation Scores

**Figure 3:**
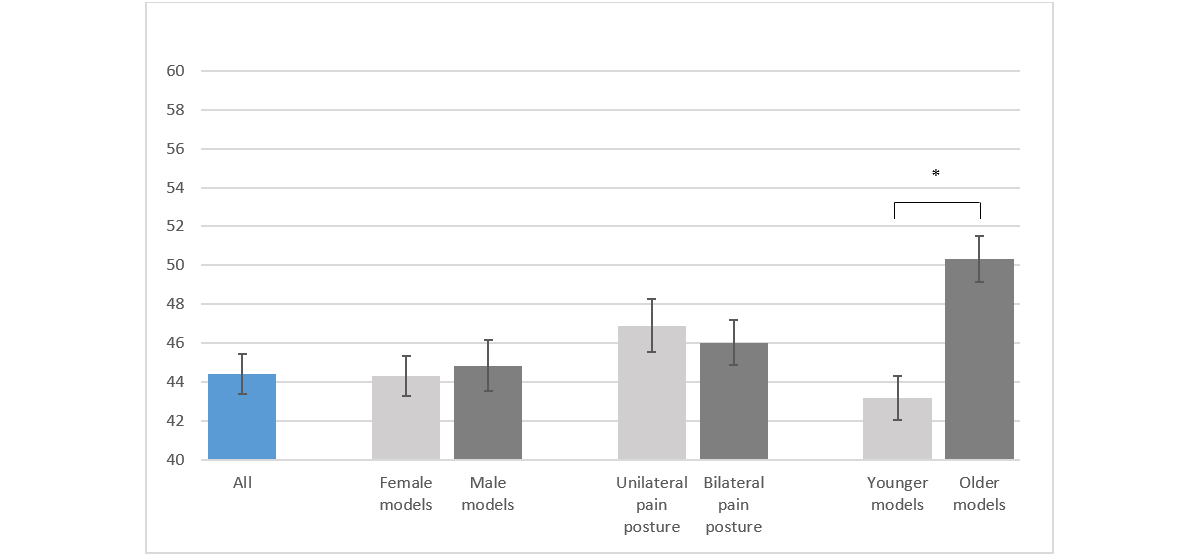
Representation scores of patients with migraine for all photos and different categories. Values are mean (SD), and the asterisk indicates *P*<.001.

When asked how much the images corresponded to their own experience of migraine, the patients answered with a mean representation score of 44.4% (SD 19.8%). Photos with older models were considered more representative than photos with younger models (mean 50.3%, SD 22.7% vs mean 43.2%, SD 21.9%; *P*<.001). The gender of the models did not lead to significant rating differences in the representation score (*P*>.99). Photos with a unilateral pain posture had similar scores as did photos with a bilateral pain posture (*P*>.99, Figure 3).

The mean representation score for all photos and in each category was significantly lower than the corresponding realism score (*P*<.001 for all categories).

There was a negative correlation between the highest level of education and both realism and representation scores: the higher the degree, the less realistic (*P*<.001, *r*=0.26) and representative (*P*<.001, *r*=0.29) the images were rated in all categories. Further analyses revealed a positive correlation between the patients’ age and the realism (*P*=.047, *r*=0.11) and representation scores (*P*=.04, *r*=0.12) of images with older models. There was no correlation with the gender or ethnicity or with the occurrence of migraine among close friends or family members.

### Health Care Workers and Students

#### Realism Scores

**Figure 4:**
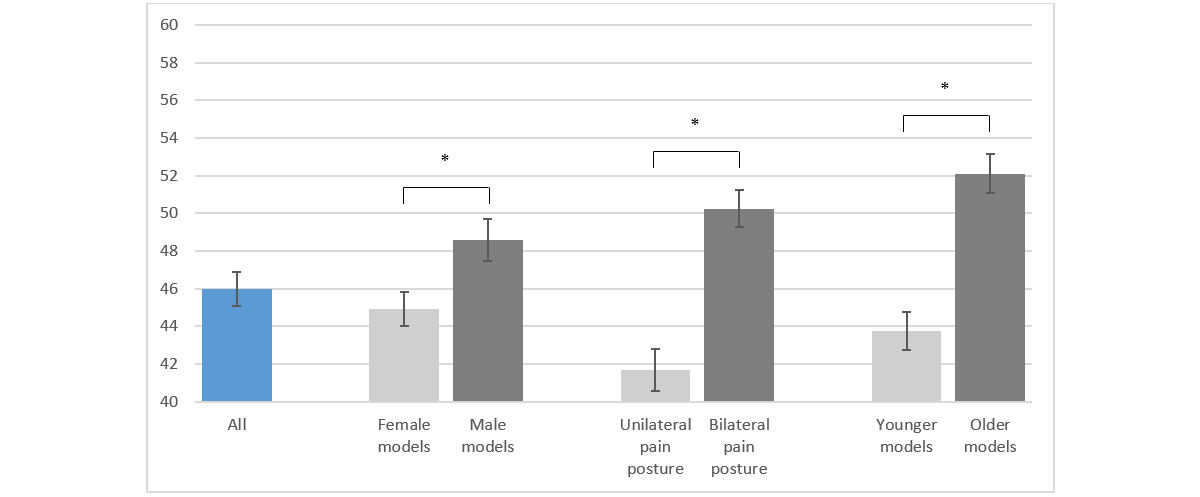
Realism scores of health care workers and students for all photos and different categories. Values are mean (SD), and the asterisk indicates *P*<.001.

In the health care group, the 10 photos had a mean realism score of 46.0% (SD 16.2%). Similar to the migraine group, only 3 photos were rated as >50% for realism. Photos with male models received higher scores than photos with female models (mean 48.6%, SD 20.2% vs mean 44.9%, SD 16.4%; *P*<.001). A bilateral pain posture was considered more realistic than a unilateral pain posture (mean 50.2%, SD 18.1% vs mean 41.7%, SD 20.0%; *P*<.001). Photos with older models were rated higher than photos with younger models (mean 52.1%, SD 18.8% vs mean 43.8%, SD 18.3%; *P*<.001). Figure 4 shows the mean realism scores in the different categories.

There was no correlation to the gender, ethnicity, or age of the participant nor to regular contact with people with migraine (*P*>.99). Further, we detected a negative correlation with the highest level of education in this group (*P*=.004, *r*=0.16).

### Comparison Between Patients With Migraine and Health Care Workers and Students

Patients with migraine rated photos with a unilateral pain posture as significantly more realistic than the health care group (*P*<.001). Overall, the mean realism score for all photos did not differ between the two groups (*P*=.23). Both groups rated photos with male and older models as more realistic than photos with female and younger models. The mean realism scores for photos with females (*P*=.20), males (*P*>.99), younger models (*P*=.14), and older models (*P*=.53) were similar between groups.

## Discussion

Patients with migraine and health care workers perceived commonly used stock photos of migraine attacks as slightly or moderately realistic. Patients identified their own migraine experience in these photos to an even lesser degree. Both groups rated photos with male and older models as more realistic than photos with female or younger ones. Among health care workers, a bilateral pain representation was considered more realistic than a unilateral pose.

The differences between media-based representations and the clinical reality have been described by Gvantseladze et al [14]. An analysis of the top 200 images under the search term “migraine” in 2 popular image-searching websites revealed that the majority of these images represented slim White females in a classic pain pose holding one or both temples [14]. The authors argued that this overrepresentation of ectomorph body types and stereotypical pain behaviors may contribute to the social stigmatization of patients with migraine [14]. Our results confirmed that not only experts but also patients doubt the realism of these images. In line with these findings, the Coalition for Headache and Migraine Patients (CHAMP) stated that media representation is often unrealistic and unlikely to display the severity of migraine [17]. The difference between the standard migraine representation and actual migraine behavior may result in the minimization of symptoms and misunderstanding of people living with this disease.

Such an example was illustrated by a social media trend from 2018, in which models and influencers published photos of themselves in the so-called “migraine pose,” touching one side of their face [18]. The fashion magazine *Elle USA* stated that this “flattering” pose “tightens the face, makes your cheekbones look more prominent, and lifts the brows” [18]. The use of the term migraine to name a glamorous pose indicates a lack of public acceptance for migraine as an extremely burdening condition. In line with the CHAMP Image Guide [17], our results support the need for a more accurate portrayal of migraine attacks. A better representation could include migraine symptoms other than headache, such as photophobia, nausea, or cognitive impairment [17]. A more diverse depiction could also help to move away from the classic temporal headache as the only accepted form of migraine pain. For example, a large proportion of migraine patients also have neck pain during attacks [19], which is almost never displayed as a feature of migraine [20].

Hospital employees and medical students shared the patients’ critical view of these images. This selected population was able to recognize that this stereotypical representation does not substantially correspond to reality. A more realistic representation of migraine attacks could also have a positive impact on patients’ treatment. The process toward effective migraine therapy is often lengthy and difficult [10]. The inaccurate, yet commonly accepted, representation of attacks could lead to a delay in the recognition and diagnosis of migraine, if patients experiencing an attack do not resemble these common depictions [10]. Giving visibility to migraine in all its facets could therefore alleviate not only social stigmatization but also the therapeutic burden.

Migraine patients and the health care group rated the laterality of the headache pose differently. A unilateral representation was perceived as more realistic by the patients. Unilaterality is one of the key migraine characteristics according to the ICHD-3 but is not a mandatory criterion for migraine diagnosis [15]. Bilateral pain occurs frequently and, in older patients, it is even more common than strict unilateral attacks [21]. Patients with migraine at a tertiary headache center like ours might be better educated about the typical characteristics of migraine and therefore rate unilaterality as a more realistic migraine feature than the nonaffected group.

Migraine prevalence is 3 times higher in women than in men [3]. However, photos with male models were considered significantly more realistic than those with female models in this analysis, regardless of the rater’s gender. This observation fits in well with the literature on gender bias, according to which pain disorders in women are taken less seriously than in men [22,23]. Pain expressions of females with chronic pain are underestimated compared to males, and women’s pain is considered less severe [24]. In the health care system, women are less likely to receive pain medication than men [25]. On the contrary, psychosocial treatments are more often recommended to female patients experiencing pain [26]. Women with pain diseases are frequently met with skepticism and have to struggle to be believed, which might lead to shame and frustration [27]. If the woman is physically attractive, the credibility of her pain is even lower, which might be applicable to our photo models [28].

Similar considerations may apply to younger patients, especially if female. Young people with pain are often perceived as less ill, based on their healthy physical appearance [29]. This might explain why photos with older models were perceived as more realistic in our survey. This is in contrast with the epidemiology of migraine, which shows a prevalence peak during young adulthood [30].

Finally, our analysis showed that less educated people rated migraine stock images as more realistic than participants with a high level of education. People with a lower education are more likely to be influenced by the media [31,32]. Therefore, it is possible that they are accustomed to this type of migraine representation and do not question its realism. This high receptivity to the media could be useful for educational programs and campaigns to raise awareness for migraine and convey a more accurate and realistic representation.

This is the first study to analyze the perception of commonly used migraine images in a large cohort of patients with migraine and health care workers. Patients with migraine were selected directly from our Headache Center, which ensured a correct diagnosis. Due to data protection regulations, patients could not be contacted by email or telephone. Given that the only possible way to contact the patient was via mail, the response rate of over 30% is within the normal range of response.

A limitation of the study is that participants completed the survey anonymously online without supervision, which might have a negative impact on data reliability. Some biases may have affected our findings: people with a pre-existing awareness of this topic might have participated to a higher extent in the survey; this applies to younger people who are frequent users of the internet as well. We also divided the photos in six categories, but not all image characteristics were taken into account. For example, the outfit of the models, the surrounding environment, or the severity of the expression of pain were not considered and may represent confounding factors. In addition, only health care workers and students were enrolled in the comparison group, which might not be entirely representative of the general population. The extension of the survey to other members of the public might provide further insights on the perception of migraine.

To conclude, the media representation of migraine was considered at best moderately realistic in our large cohort of patients with migraine and health care workers. The rating of male and older models as more realistic contradicts migraine epidemiology. A more truthful representation of migraine is needed in order to raise awareness of the burden of this disease and to reduce migraine-related social stigma.

## Abbreviations

CHAMP: Coalition for Headache and Migraine Patients
ICHD-3: International Classification of Headache Disorders–3
REDCap: Research Electronic Data Capture
